# Segment-specific intestinal bacterial community structure is associated with short-chain fatty acid profiles and mucosal morphology in two high-altitude sheep breeds

**DOI:** 10.3389/fmicb.2026.1873761

**Published:** 2026-07-09

**Authors:** Youji Ma, Dengpan Li, Zhanjing Liu, Xujie Li

**Affiliations:** College of Animal Science and Technology, Gansu Agricultural University, Lanzhou, China

**Keywords:** bacterial community, full-length 16S rRNA gene sequencing, high-altitude sheep, intestinal niche, mucosal morphology, short-chain fatty acids

## Abstract

The gastrointestinal tract of grazing ruminants contains segment-specific microbial niches that contribute to fermentation metabolism and intestinal function. However, the intestinal bacterial communities and their relationships with fermentation products and mucosal morphology remain insufficiently characterized in alpine sheep breeds. In this study, Tianhua mutton sheep and Gansu Alpine fine-wool sheep maintained under the same grazing conditions were compared using the techniques of hematoxylin and eosin (H&E) staining, gas chromatography, and full-length 16S rRNA sequencing. Histological analysis showed that Tianhua mutton sheep had lower crypt depth and higher villus-height-to-crypt-depth ratios in the duodenum and ileum, while muscular layer thickness differed between breeds in a segment-dependent manner. Short-chain fatty acids (SCFAs) analysis revealed breed-related differences in specific intestinal segments, with Tianhua mutton sheep showing higher levels of several major SCFAs components and total SCFA in the duodenum, jejunum, cecum, and colon. Full-length 16S rRNA sequencing demonstrated distinct bacterial community structures between the two breeds and clear spatial variation along the intestinal tract. Tianhua mutton sheep exhibited higher bacterial richness in several segments and enrichment of Firmicutes and *Christensenellaceae_R_7_group*, whereas Gansu Alpine fine-wool sheep showed higher abundances of Bacteroidota, Verrucomicrobiota, and *Akkermansia* in specific segments. Functional prediction indicated that KEGG level 3 differences were mainly concentrated in the cecum and colon, involving pathways related to microbial metabolism, carbon metabolism, ABC transporters, and two-component systems. Spearman correlation analysis further linked selected genera with SCFA traits, whereas fewer associations were observed with small-intestinal histomorphological indices. These findings suggest that Tianhua mutton sheep and Gansu Alpine fine-wool sheep differ in intestinal morphology, fermentation characteristics, and microbial structure and function, providing new information for understanding breed-specific host–microbiota interactions in alpine grazing sheep.

## 1 Introduction

The sustainable development of animal husbandry in alpine pastoral regions presents a significant challenge within global agriculture. These ecosystems, characterized by low oxygen availability, extreme temperature fluctuations, and seasonal variations in forage quality, demand unique adaptations from livestock species. Enhancing breed characteristics is paramount for increasing productivity while maintaining the delicate ecological balance of these vulnerable areas (Jarque-Bascuñana et al., 2022). Successful adaptation of ruminants involves complex physiological adjustments, particularly in the gastrointestinal system, the primary interface for nutrient extraction from scarce and fibrous feed resources. Gansu alpine fine-wool sheep represent a valuable genetic resource exemplifying remarkable adaptation to these demanding environments. Developed in the 1980s for the high-altitude pastures of the Qilian Mountains, this breed demonstrates exceptional resilience to alpine conditions (Huang et al., 2017) and produces superior quality wool (Chen et al., 2024). In response to evolving market demands, Tianhua mutton sheep were developed in the 21st century as an innovative dual-purpose breed. This breeding initiative integrated the wool characteristics and environmental resilience of Gansu alpine fine-wool sheep with enhanced meat production capabilities from the South African Meat Merino lineage (Li et al., 2024; Liu et al., 2022). The phenotypic differences between these two breeds, particularly in production traits, suggest underlying physiological and metabolic variations that warrant detailed investigation (Ma et al., 2025; Li et al., 2024).

The intestinal barrier in ruminants serves as a crucial interface between the organism and its environment, performing essential functions in nutrient absorption, pathogen exclusion, and immunological regulation (Zhang et al., 2024; Li et al., 2023). This sophisticated biological system maintains intestinal homeostasis through multiple coordinated mechanisms, including physical barriers formed by epithelial cells and tight junctions, chemical barriers comprising mucus and antimicrobial peptides, and immunological barriers involving specialized immune cells and their secretions (Martel et al., 2022). The integrity of this complex barrier system is fundamental to animal health, as its compromise can lead to serious consequences including impaired nutrient assimilation, reduced immunity, metabolic disorders, and increased susceptibility to infectious diseases (Casas et al., 2020; Paone and Cani, 2020). In high-altitude environments where metabolic efficiency is paramount, optimal intestinal barrier function becomes even more critical for survival and productivity. Evaluation of intestinal health typically involves comprehensive histomorphology assessment using parameters such as villus height, crypt depth, and the villus-to-crypt ratio, commonly measured through hematoxylin-eosin staining techniques (Munoz et al., 2023). These morphological indicators provide valuable insights into the functional capacity of the intestinal epithelium for nutrient absorption and barrier maintenance, with greater villus height and favorable villus-to-crypt ratios generally indicating enhanced absorptive surface area and functional efficiency (Rubin and Levin, 2016). Additionally, short-chain fatty acids, produced through microbial fermentation of dietary fibers, play a pivotal role in strengthening the intestinal chemical barrier by stimulating mucin production through MUC2 gene expression and modulating local immune responses (Dalile et al., 2019; Fan et al., 2021). These microbial metabolites also serve as important energy sources for colonocytes and contribute to the maintenance of optimal intestinal pH, further enhancing barrier function and overall gut health (Pearce et al., 2020).

The gut microbiota comprises a diverse ecosystem of microorganisms that profoundly influence host physiology through multiple mechanisms, particularly in ruminants where microbial fermentation is central to energy extraction (Culp and Goodman, 2023). Beneficial bacterial species, including Bifidobacterium and Lactobacillus, enhance barrier function by competitive exclusion of pathogens, production of antimicrobial compounds, and stimulation of mucosal immunity (Martel et al., 2022; Erttmann et al., 2022). These commensal organisms form complex biofilms that reinforce the intestinal mucus layer and promote epithelial integrity, while also engaging in cross-feeding relationships that optimize metabolic output (Culp and Goodman, 2023). Specific taxa such as Christensenellaceae have been identified as heritable components of the gut microbiome associated with host health status (Waters and Ley, 2019), while Akkermansia muciniphila has emerged as a next-generation beneficial microorganism crucial for mucus barrier integrity (Cani et al., 2022). Conversely, microbial dysbiosis characterized by overgrowth of pathogenic species or reduction in beneficial taxa can disrupt intestinal homeostasis, damage villus architecture, and trigger inflammatory responses (Budden et al., 2017; El-Saadony et al., 2021). Such disruptions often lead to impaired nutrient absorption, reduced growth performance, and increased incidence of metabolic diseases, ultimately compromising the animal's ability to thrive in challenging environments. Multiple factors influence the composition and function of the gut microbiota in ruminants, with dietary composition significantly affecting microbial communities (Tao et al., 2017; Gao et al., 2024). Additionally, host genetic factors play a crucial role in shaping microbial ecosystems, as evidenced by significant breed-related variations observed among different sheep breeds including Hu, Tan, and Dorper sheep (Cheng et al., 2022; Yang et al., 2022). These genetic influences operate through multiple mechanisms, including differences in immune function, mucosal architecture, and nutrient metabolism that collectively create distinct ecological niches for microbial colonization (Zhao et al., 2023).

Recent research has increasingly highlighted the role of gut microbiota in host adaptation to challenging environments. Studies on Tibetan wild asses (Liu et al., 2022), Tibetan pigs (Zhao et al., 2023), and small wild ruminants in the Three-River-Source National Park (Liu et al., 2024) have reported environment-associated microbial features under high-altitude conditions. In sheep, distinct bacterial communities have been observed between Tibetan sheep and Gansu Alpine fine-wool sheep grazing on the Qinghai-Tibetan Plateau (Huang et al., 2017), and differences in rumen development between Tianhua mutton sheep and Gansu Alpine fine-wool sheep under grazing conditions have also been documented (Li et al., 2024). Building on these findings, the present study compared intestinal bacterial communities, SCFA profiles, and small-intestinal mucosal morphology between Tianhua mutton sheep and Gansu Alpine fine-wool sheep raised under the same alpine grazing conditions. We hypothesized that breed-related differences in intestinal microbiota and fermentation profiles would be associated with differences in small-intestinal mucosal morphology and segment-specific microbial functions. This study provides information for understanding gut microbial ecology and host–microbiota interactions in these two alpine sheep breeds.

## 2 Materials and methods

### 2.1 Ethics statement

All experimental protocols were conducted in compliance with the Guidelines for the Care and Use of Laboratory Animals established by the Ministry of Science and Technology of China (Approval No. 2006-398) and were reviewed and approved by the Animal Ethics Committee of Gansu Agricultural University (Approval No. GSAU-AEW-2020-0057). All procedures adhered to ethical standards for animal welfare.

### 2.2 Test animals and experimental design

The study was conducted in a natural high-altitude pasture in Duolong Village, Tianzhu County, Gansu Province, China, at approximately 2,800 m above sea level. The pasture was mainly composed of *Polygonum viviparum* and *Elymus nutans*, which together represented the dominant available herbage, and its nutrient composition is shown in Supplementary Table 1. Tianhua mutton sheep and Gansu Alpine fine-wool sheep were raised together under the same local traditional grazing management system. The lambs were identified by ear tags at approximately 30 d of age, suckled their dams during early life, gradually followed the ewe flock to graze on natural pasture, and continued to graze together with the same local flock after weaning, with no concentrate supplementation and free access to water. A total of 12 clinically healthy 6-month-old female lambs born during the same lambing period were selected from the mixed grazing flock for intestinal sample collection, including six Tianhua mutton sheep and six Gansu Alpine fine-wool sheep. Before slaughter, the animals were fasted for 12 h and deprived of water for 2 h, electrically stunned under veterinary supervision, and humanely slaughtered in accordance with approved ethical protocols. After slaughter, contents from the duodenum, jejunum, ileum, cecum, and colon were collected for SCFA**s** analysis and 16S rRNA gene sequencing. Small-intestinal tissues from the duodenum, jejunum, and ileum were fixed in 4% paraformaldehyde for H&E staining.

### 2.3 HE staining

To evaluate small-intestinal histomorphology, tissue samples from the middle of the duodenum, jejunum, and ileum were fixed in 4% paraformaldehyde, embedded in paraffin, and sectioned at a thickness of 5 μm. Following H&E staining according to standard protocols, the morphological structures were examined under a light microscope. Digital images were captured at 5 × magnification. Villus height, crypt depth, and muscular layer thickness were measured in five well-oriented, randomly selected fields per sample using Image-Pro Plus 6.0 software.

### 2.4 Determination of SCFAs

SCFAs concentrations were determined by gas chromatography (GC) (Agilent 6890N, USA). Before centrifugation, cecal and colonic content samples were diluted 1:1 with ddH_2_O and kept overnight at 4 °C, and then processed together with small-intestinal content samples from the duodenum, jejunum, and ileum. All intestinal content samples were centrifuged at 5,000 rpm for 10 min at 4 °C. The supernatant was then derivatized: a 1 mL aliquot was mixed with 0.2 mL of 25% (w/v) metaphosphoric acid solution containing 2-ethylbutyric acid (2EB; 2 g/L) as an internal standard. The mixture was vortexed, kept on ice for 30 min, and centrifuged again at 8,000 rpm for 10 min. The resulting supernatant was filtered through a 0.22 μm hydrophobic PTFE membrane into a 2 mL amber vial and stored at −20 °C until GC analysis. Separation was performed on an HP-INNOWAX capillary column (19091N-213, Agilent, USA) with the following temperature program: hold at 60 °C for 2 min, ramp to 140 °C at 10 °C/min, then ramp to 170 °C at 3 °C/min. The injector and detector temperatures were set to 250 °C and 280 °C, respectively.

### 2.5 Microbial DNA extraction

For full-length 16S rRNA gene sequencing, intestinal content samples from all 12 slaughtered lambs were used for microbial community analysis. Samples were collected separately from five intestinal segments, including the duodenum, jejunum, ileum, cecum, and colon, resulting in 60 samples in total. Samples from Tianhua mutton sheep were assigned to the T group, whereas samples from Gansu Alpine fine-wool sheep were assigned to the G group. For segment-specific analysis, duodenal, jejunal, ileal, cecal, and colonic samples from Tianhua mutton sheep were labeled as Tdu1–Tdu6, Tje1–Tje6, Til1–Til6, Tce1–Tce6, and Tco1–Tco6, respectively; the corresponding samples from Gansu Alpine fine-wool sheep were labeled as Gdu1–Gdu6, Gje1–Gje6, Gil1–Gil6, Gce1–Gce6, and Gco1–Gco6, respectively. Microbial genomic DNA was extracted using the TGuide S96 Magnetic Bead Soil/Fecal DNA Extraction Kit (Tiangen Biotech, China) according to the manufacturer's instructions. The extracted DNA was eluted with AE buffer. DNA purity and concentration were assessed using a NanoDrop spectrophotometer (Thermo Fisher Scientific, USA), and DNA integrity was evaluated by 1% agarose gel electrophoresis and LabChip analysis. Samples that met the quality criteria were used for downstream amplification and sequencing.

### 2.6 Full-length 16S rRNA sequencing

Qualified DNA samples were subjected to full-length 16S rRNA gene amplification targeting the V1–V9 hypervariable regions. The primers used were 27F (AGRGTTTGATYNTGGCTCAG) and 1492R (TASGGHTACCTTGTTASGACTT), both tailed with sample-specific barcode sequences for multiplexed PacBio sequencing. Amplification products were purified, quantified, normalized, and used to construct SMRT Bell libraries. Library quality was verified using an Agilent 2,100 Bioanalyzer. Qualified libraries were sequenced on the PacBio Sequel II platform (Pacific Biosciences, USA) by Biomarker Technologies Corporation (Beijing, China).

### 2.7 Bioinformatic processing of sequencing data

Raw sequencing data in BAM format were processed using SMRT Link software (v8.0) to generate circular consensus sequences (CCS) with a minimum of three full passes. After demultiplexing based on barcode sequences and trimming of primers, sequences underwent strict length filtering (1,200–1,800 bp) and chimera removal with UCHIME algorithm in USEARCH (v10.0). High-quality sequences were clustered into OTUs at 97% similarity threshold using UPARSE pipeline. Taxonomic assignment was performed in QIIME2 with a Naive Bayes classifier trained on the SILVA 138.1 database at 70% confidence threshold. Alpha diversity indices (Ace, Chao1, Shannon, Simpson) were calculated with rarefaction curves generated to evaluate sequencing depth. Beta diversity was assessed using Bray–Curtis distance metric and visualized through PCoA and NMDS analyses. Microbial community composition was summarized at the phylum and genus levels. LEfSe analysis was performed to identify differentially abundant taxa between groups using the non-parametric Kruskal–Wallis test, with *P* < 0.05 and an LDA score > 4.0 considered significant. PICRUSt2 (v2.3.0) was used to predict microbial functions, and KEGG level 3 pathways were used for downstream analysis. The top 15 predicted KEGG level 3 pathways in each intestinal segment were visualized as bar plots. Differential KEGG level 3 pathways between the two breeds were compared within each segment; pathways with BH-adjusted *P* < 0.05 were considered significant. For segments without significant differences, the top 10 pathways ranked by adjusted *P* values were shown.

### 2.8 Statistical analysis

Data were organized in Microsoft Excel 2016 and analyzed using SPSS Statistics v26.0 and R software v4.3.2. The individual lamb was used as the experimental unit. For SCFA concentrations and intestinal histomorphological indices, normality and homogeneity of variance were assessed using the Shapiro–Wilk and Levene's tests, respectively, and independent-samples *t*-tests, Welch's *t*-tests, or Mann–Whitney U tests were used as appropriate. Alpha-diversity indices were analyzed using non-parametric tests; Mann–Whitney U tests were used for comparisons between breeds, whereas Friedman tests followed by pairwise Wilcoxon signed-rank tests were used for comparisons among the five intestinal segments within each breed. *P*-values from *post hoc* comparisons, KEGG level 3 functional comparisons, and Spearman correlation analyses were adjusted using the Benjamini–Hochberg (BH) method. BH-adjusted *P* < 0.05 was considered statistically significant. Spearman correlations between the top 20 genera and phenotypic traits were visualized using the R packages ggplot2 v3.5.1, rstatix v0.7.2, and pheatmap v1.0.12.

## 3 Results

### 3.1 Morphological differences in the small intestine

As shown in Table 1 and Supplementary Figure 1, in the duodenum and ileum, the crypt depth of Tianhua mutton sheep was significantly lower than that of Gansu Alpine fine-wool sheep, whereas the villus height-to-crypt depth ratio (V/C) was significantly higher in Tianhua mutton sheep (*P* < 0.05). For muscular layer thickness, Gansu Alpine fine-wool sheep showed a significantly greater value in the duodenum, while Tianhua mutton sheep showed a significantly greater value in the ileum (*P* < 0.05). In the jejunum, no significant differences were observed between the two breeds in crypt depth, muscular layer thickness, villus height, or V/C.

**Table 1:** Comparison of small intestinal morphology between Tianhua Mutton Sheep and Gansu Alpine fine wool sheep.

Intestinal segment	Item	Tianhua mutton sheep	Gansu alpine fine wool sheep	*P*
Duodenum	Villus height/ μm	508.50 ± 62.13	507.50 ± 56.95	0.98
	Crypt depth/ μm	192.17 ± 42.49^b^	250.83 ± 38.20^a^	0.03
	V/C	2.74 ± 0.81^a^	2.06 ± 0.40^b^	0.04
	Muscular layer thickness/ μm	224.17 ± 22.32^b^	273.00 ± 40.35^a^	0.03
Jejunum	Villus height/ μm	395.33 ± 56.93	388.00 ± 57.89	0.83
	Crypt depth/ μm	251.83 ± 54.62	244.67 ± 51.40	0.82
	V/C	1.66 ± 0.54	1.65 ± 0.45	0.98
	Muscular layer thickness/ μm	226.67 ± 24.15	198.00 ± 25.29	0.07
Ileum	Villus height/ μm	583.33 ± 66.16	522.83 ± 70.29	0.16
	Crypt depth/ μm	289.17 ± 17.19^b^	320.17 ± 21.42^a^	0.02
	V/C	2.02 ± 1.28^a^	1.63 ± 0.34^b^	0.04
	Muscular layer thickness/ μm	261.83 ± 47.15^a^	199.50 ± 48.13^b^	0.04

Values are presented as mean ± SD. Different superscript letters within the same row indicate significant differences between breeds (*P* < 0.05) (*n* = 12).

### 3.2 Short-chain fatty acid profiles in intestinal contents

Several SCFA indices differed between the two breeds in specific intestinal segments (Table 2). In the duodenum, Tianhua mutton sheep had higher acetic acid, propionic acid, butyric acid, total SCFA, and A:P than Gansu Alpine fine-wool sheep (*P* < 0.05). In the jejunum, acetic acid, propionic acid, and total SCFA were higher in Tianhua mutton sheep (*P* < 0.05). In the cecum, acetic acid, butyric acid, and total SCFA were higher in Tianhua mutton sheep, while in the colon, propionic acid and total SCFA were higher in Tianhua mutton sheep (*P* < 0.05). No significant breed-related differences were observed for the remaining SCFA indices within each intestinal segment.

**Table 2:** Short-chain fatty acid profiles in intestinal contents of Tianhua mutton sheep and Gansu Alpine fine-wool sheep.

Intestinal segment	Item	Tianhua mutton sheep	Gansu alpine fine wool sheep	*P*
Duodenum	Acetic acid, mmol/L	36.62 ± 1.12^a^	22.29 ± 3.94^b^	< 0.01
	Propionic acid, mmol/L	8.29 ± 1.04^a^	6.93 ± 0.60^b^	0.02
	Butyric acid, mmol/L	4.04 ± 0.33^a^	3.14 ± 0.79^b^	0.04
	Total SCFA, mmol/L	52.39 ± 1.44^a^	35.26 ± 4.95^b^	< 0.01
	A:P	4.47 ± 0.53^a^	3.23 ± 0.61^b^	< 0.01
Jejunum	Acetic acid, mmol/L	12.24 ± 0.92^a^	7.12 ± 0.15^b^	< 0.01
	Propionic acid, mmol/L	3.64 ± 0.45^a^	2.58 ± 0.58^b^	0.01
	Butyric acid, mmol/L	1.32 ± 0.48	1.29 ± 0.07	0.87
	Total SCFA, mmol/L	18.27 ± 1.65^a^	11.90 ± 0.38^b^	< 0.01
	A:P	3.39 ± 0.22	2.87 ± 0.64	0.11
Ileum	Acetic acid, mmol/L	15.26 ± 1.82	13.44 ± 0.15	0.06
	Propionic acid, mmol/L	5.75 ± 1.56	4.69 ± 0.58	0.17
	Butyric acid, mmol/L	3.62 ± 0.69	3.56 ± 0.75	0.89
	Total SCFA, mmol/L	27.46 ± 3.26	24.46 ± 1.54	0.07
	A:P	2.74 ± 0.42	2.90 ± 0.37	0.50
Cecum	Acetic acid, mmol/L	25.58 ± 0.92^a^	23.46 ± 1.40^b^	0.01
	Propionic acid, mmol/L	6.94 ± 0.65	6.88 ± 0.43	0.86
	Butyric acid, mmol/L	6.15 ± 0.27^a^	5.66 ± 0.23^b^	0.01
	Total SCFA, mmol/L	40.84 ± 1.24^a^	37.76 ± 1.73^b^	0.01
	A:P	3.72 ± 0.40	3.42 ± 0.21	0.13
Colon	Acetic acid, mmol/L	12.40 ± 0.29	12.00 ± 0.58	0.17
	Propionic acid, mmol/L	6.52 ± 0.26^a^	6.00 ± 0.45^b^	0.04
	Butyric acid, mmol/L	2.73 ± 0.11	2.50 ± 0.23	0.06
	Total SCFA, mmol/L	23.54 ± 0.36^a^	22.09 ± 1.28^b^	0.04
	A:P	1.91 ± 0.10	2.00 ± 0.06	0.11

Values are presented as mean ± SD. Different superscript letters within the same row indicate significant differences between breeds (*P* < 0.05) (*n* = 12).

### 3.3 Gut microbiota differences between Tianhua mutton sheep and Gansu Alpine fine wool sheep

**Figure 1:**
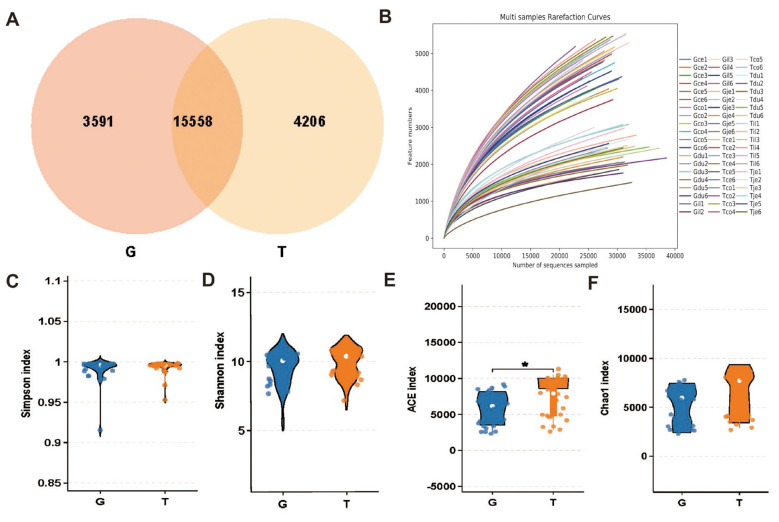
Comparative analysis of gut microbial diversity between the two distinct sheep breeds: Tianhua mutton (T group) and Gansu Alpine fine wool (G group). **(A)** Venn diagram showing the OTUs shared in the gut between the T and G groups. **(B)** Rarefaction curves of all sequenced samples. du, duodenum; je, jejunum; il, ileum; ce, cecum; co, colon. **(C–F)** Simpson, Shannon, ACE and Chao1 indices of bacterial communities in the T and G groups, respectively. T group, Tianhua mutton sheep; G group, Gansu Alpine fine-wool sheep. Asterisks indicate significant differences between breeds based on Mann–Whitney U tests with BH correction.

**Figure 2:**
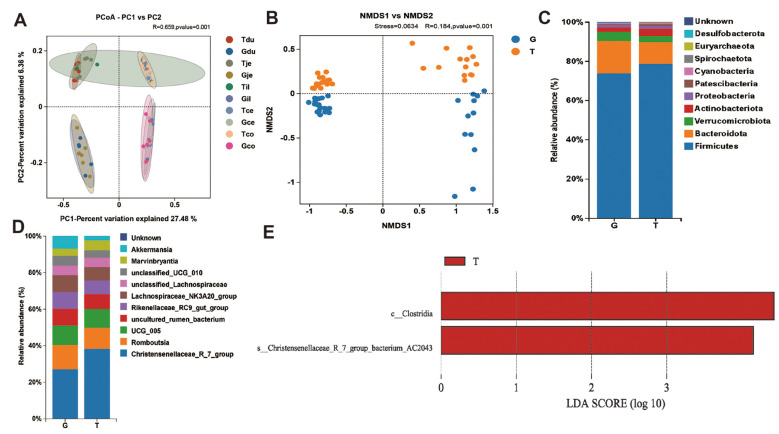
Comparative analysis of intestinal microbiota between Tianhua mutton sheep and Gansu Alpine fine-wool sheep. **(A)** PCoA based on Bray–Curtis distances across all intestinal samples. du, duodenum; je, jejunum; il, ileum; ce, cecum; co, colon. **(B)** NMDS based on Bray–Curtis distances across all intestinal samples. **(C)** Phylum-level bacterial composition. **(D)** Genus-level bacterial composition. **(E)** LEfSe analysis of differential taxa between the T and G groups. Differential taxa were identified using an LDA score > 4.0 and *P* < 0.05. T group, Tianhua mutton sheep; G group, Gansu Alpine fine-wool sheep.

The gut microbiota was characterized and compared throughout the intestinal tract of both sheep breeds. Analysis revealed 15,558 shared microbial features between the two breeds (Figure 1A). Rarefaction analysis was further evaluated together with sequencing quality metrics (Figure 1B; Supplementary Table 2). Across all samples, effective CCS reads ranged from 27,272 to 38,973, and Good's coverage values ranged from 0.8880 to 0.9830, with an average of 0.9451, indicating that the sequencing depth captured the majority of bacterial diversity for downstream comparative analysis. Alpha-diversity analysis demonstrated a significantly higher Ace index in Tianhua mutton sheep (*P* < 0.05), with no significant differences observed in other diversity indices (Figures 1C–F). β-Diversity analysis using two distinct methods revealed significant separation between the microbial communities of the two breeds (Figures 2A, B). Both breeds exhibited similar spatial organization along the intestinal tract, with duodenal and jejunal samples clustering together, and cecal and colonic samples forming a distinct cluster. At both phylum and genus levels, Tianhua mutton sheep showed increased relative abundances of Firmicutes and *Christensenellaceae_R_7_group*, but reduced abundances of Bacteroidota, Verrucomicrobiota, and *Romboutsia* compared to Gansu Alpine fine wool sheep (Figures 2C, D). LEfSe analysis identified two significantly enriched taxa in Tianhua mutton sheep: *Clostridia* and *Christensenellaceae-R-7-group-bacterium-AC2043* (Figure 2E).

### 3.4 Segmental variations in gut microbiota structure

**Figure 3:**
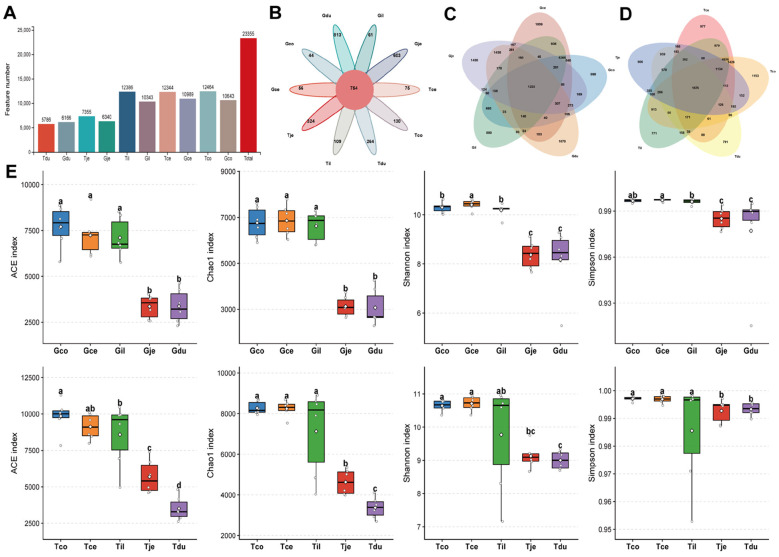
Microbial composition across intestinal segments in Gansu Alpine fine-wool sheep and Tianhua mutton sheep. **(A)** Distribution of microbial feature counts across intestinal segments. T, Tianhua mutton sheep; G, Gansu Alpine fine-wool sheep; du, duodenum; je, jejunum; il, ileum; ce, cecum; co, colon. **(B)** Venn diagram showing shared and unique microbial features among intestinal segments. **(C)** Feature overlap across five intestinal segments in Gansu Alpine fine-wool sheep. **(D)** Feature overlap across five intestinal segments in Tianhua mutton sheep. **(E)** Alpha-diversity indices of intestinal microbiota in five intestinal segments of both sheep breeds. ACE, Chao1, Shannon and Simpson indices are shown from left to right, with Gansu Alpine fine-wool sheep in the upper panels and Tianhua mutton sheep in the lower panels. Different lowercase letters indicate significant differences among intestinal segments within the same breed based on Friedman tests followed by pairwise Wilcoxon signed-rank tests with BH correction.

Microbiota profiling across intestinal segments identified a total of 23,355 microbial features. The large intestine, including the cecum and colon, showed greater feature richness than the small intestine, including the duodenum, jejunum and ileum, in both sheep breeds (Figure 3A). Shared microbial features were observed between the same intestinal segments of the two breeds (Figure 3B), as well as among different intestinal segments within each breed (Figures 3C, D). Alpha-diversity analysis further revealed clear segmental variation along the intestinal tract (Figure 3E). In both Tianhua mutton sheep and Gansu Alpine fine-wool sheep, the ACE, Chao1, Shannon and Simpson indices were significantly lower in the duodenum and jejunum than in the ileum, cecum and colon (*P* < 0.05).

### 3.5 Interspecific comparison of alpha diversity by intestinal segment

Comparison of α-diversity between the two sheep groups within each intestinal segment showed no significant differences in the duodenum and ileum for any of the diversity indices (Chao1, Simpson, ACE, and Shannon) between Tianhua mutton sheep and Gansu Alpine fine wool sheep (Table 3). Significant differences were observed in the jejunum, cecum, and colon, where Tianhua mutton sheep exhibited higher values for Chao1, ACE, and Shannon indices (*P* < 0.05) compared to Gansu Alpine fine wool sheep.

**Table 3:** Analysis of α diversity in each intestinal segment of two sheep.

Intestinal segment	item	Tianhua mutton sheep	Gansu alpine fine wool sheep	*P*
Duodenum	ACE	3,514.54 ± 832.15	3,372.79 ± 924.32	0.91
	Chao1	3,367.67 ± 522.04	3,017.53 ± 796.42	0.49
	Simpson	0.99 ± 0.01	0.99 ± 0.03	0.12
	Shannon	9.00 ± 0.28	8.15 ± 1.38	0.20
Jejunum	ACE	5,699.97 ± 1,156.18^a^	3,360.11 ± 639.22^b^	0.01
	Chao1	4,628.77 ± 614.77^a^	3,126.92 ± 430.58^b^	0.01
	Simpson	0.99 ± 0.01	0.98 ± 0.01	0.12
	Shannon	9.13 ± 0.36^a^	8.37 ± 0.56^b^	0.04
Ileum	ACE	8,591.75 ± 2,165.25	7,103.50 ± 1,090.59	0.20
	Chao1	7,130.87 ± 2,125.37	6,622.69 ± 653.93	0.49
	Simpson	0.99 ± 0.02	1.00 ± 0.01	1.00
	Shannon	9.77 ± 1.63	10.19 ± 0.27	0.49
Cecum	ACE	9,119.14 ± 878.58^a^	7,242.02 ± 1,119.69^b^	0.04
	Chao1	8,245.33 ± 395.47^a^	6,861.69 ± 670.91^b^	0.02
	Simpson	1.00 ± 0.01	1.00 ± 0.01	1.00
	Shannon	10.71 ± 0.22	10.41 ± 0.21	0.09
Colon	ACE	9,851.07 ± 1,132.62^a^	7,704.53 ± 1,140.78^b^	0.04
	Chao1	8,278.45 ± 316.30^a^	6,761.01 ± 688.59^b^	0.01
	Simpson	1.00 ± 0.01	1.00 ± 0.01	0.74
	Shannon	10.64 ± 0.17	10.30 ± 0.22	0.09

Values are presented as mean ± SD. Different superscript letters within the same row indicate significant differences between breeds after BH correction.

### 3.6 Microbial composition of the small intestine

#### 3.6.1 Duodenal microbiota

**Figure 4:**
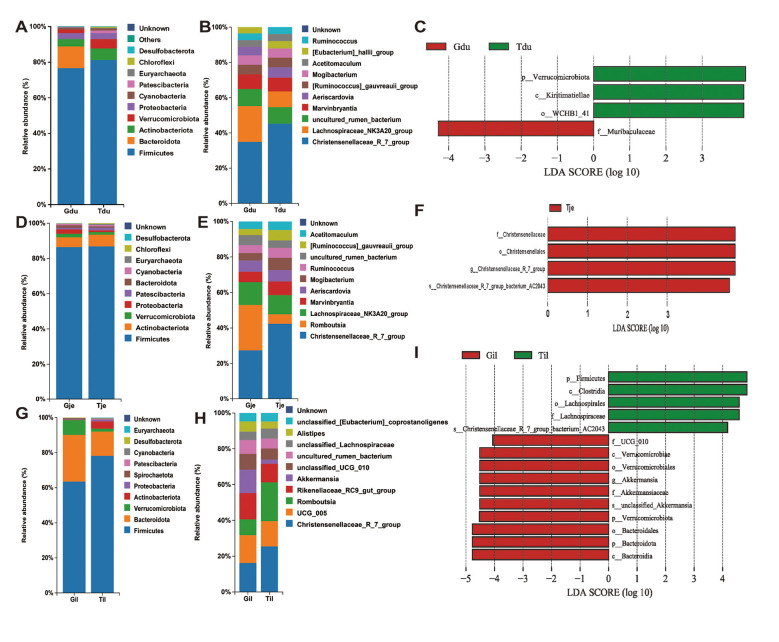
Microbial composition in the small intestine of Tianhua mutton sheep and Gansu Alpine fine-wool sheep. **(A–C)** Phylum-level composition, genus-level composition and LEfSe analysis of differential taxa in the duodenum. **(D–F)** Phylum-level composition, genus-level composition and LEfSe analysis of differential taxa in the jejunum. **(G–I)** Phylum-level composition, genus-level composition and LEfSe analysis of differential taxa in the ileum. Differential taxa were identified using an LDA score > 4.0 and *P* < 0.05. T, Tianhua mutton sheep; G, Gansu Alpine fine-wool sheep; du, duodenum; je, jejunum; il, ileum.

Duodenal microbial profiling (Figures 4A–C) revealed that Tianhua mutton sheep exhibited increased relative abundances of Firmicutes, Actinobacteriota, and *Christensenellaceae_R_7_group*. In contrast, Gansu alpine fine wool sheep showed greater proportions of Bacteroidota and *Lachnospiraceae_NK3A20_group*. LEfSe analysis identified Verrucomicrobiota, *Kiritimatiella*, and *WCHB1–41* as discriminant taxa for Tianhua mutton sheep, whereas *Muribaculaceae* served as a discriminative feature for Gansu alpine fine wool sheep.

#### 3.6.2 Jejunal microbiota

Within the jejunum (Figures 4D-F), Tianhua mutton sheep again demonstrated higher abundances of Firmicutes, Actinobacteriota, and *Christensenellaceae_R_7_group*. Conversely, Gansu alpine fine wool sheep were characterized by elevated levels of *Romboutsia* and *Lachnospiraceae_NK3A20_group*. LEfSe results indicated *Christensenellaceae* and related taxa (*Christensenellaceae-R-7-group-bacterium-AC2043* and *Christensenellales*) as biomarkers significantly associated with Tianhua mutton sheep.

#### 3.6.3 Ileal microbiota

Ileal microbial analysis (Figures 4G–I) showed Firmicutes and *Christensenellaceae_R_7_group* were more abundant in Tianhua mutton sheep, while Gansu alpine fine wool sheep possessed higher levels of Bacteroidota and *UCG*−*005*. LEfSe revealed 15 differentially abundant taxa, with *Firmicutes* distinguishing Tianhua mutton sheep, and Verrucomicrobiota, Bacteroidota (phylum level), and *Akkermansia* (genus level) characterizing Gansu alpine fine wool sheep.

### 3.7 Microbial composition of the large intestine

#### 3.7.1 Cecal microbiota

**Figure 5:**
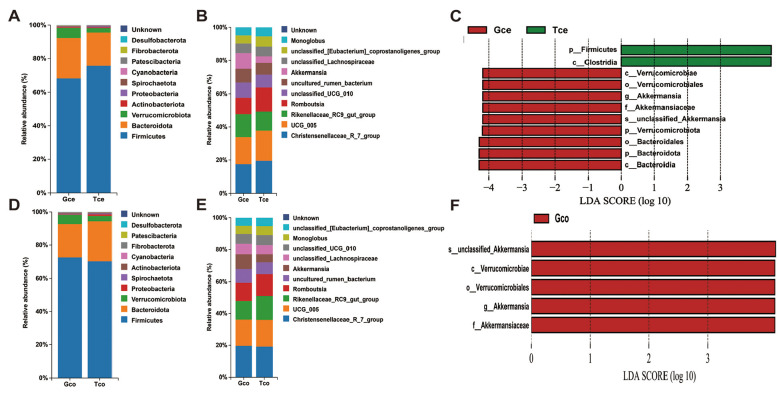
Microbial composition in the large intestine of Tianhua mutton sheep and Gansu Alpine fine-wool sheep. **(A–C)** Phylum-level composition, genus-level composition and LEfSe analysis of differential taxa in the cecum. **(D–F)** Phylum-level composition, genus-level composition and LEfSe analysis of differential taxa in the colon. Differential taxa were identified using an LDA score > 4.0 and *P* < 0.05. T, Tianhua mutton sheep; G, Gansu Alpine fine-wool sheep; du, duodenum; ce, cecum; co, colon.

Analysis of cecal microbiota (Figures 5A–C) indicated higher relative abundances of Firmicutes, *Christensenellaceae_R_7_group*, and *UCG*−*005* in Tianhua mutton sheep compared to Gansu alpine fine wool sheep. Conversely, Gansu alpine fine wool sheep showed increased relative abundance of Bacteroidota. LEfSe analysis identified 11 differentially abundant taxa, with Firmicutes demonstrating higher abundance in Tianhua mutton sheep. Verrucomicrobiota and Bacteroidota were more abundant in Gansu alpine fine wool sheep at the phylum level, while *Akkermansia* showed higher abundance at the genus level.

#### 3.7.2 Colon microbiota

In the colon (Figures 5D–F), Tianhua mutton sheep exhibited greater relative abundances of Bacteroidota, Proteobacteria, *Rikenellaceae_RC9_gut_group*, and *Romboutsia* relative to Gansu alpine fine wool sheep. The latter showed elevated abundance of Verrucomicrobiota, and *Akkermansia*. LEfSe analysis showed that Gansu alpine fine-wool sheep had higher abundances of two microbial taxa, *Verrucomicrobiae* and *Akkermansia*, compared to Tianhua mutton sheep.

### 3.8 Predicted KEGG functions and microbial associations with phenotypic traits

**Figure 6:**
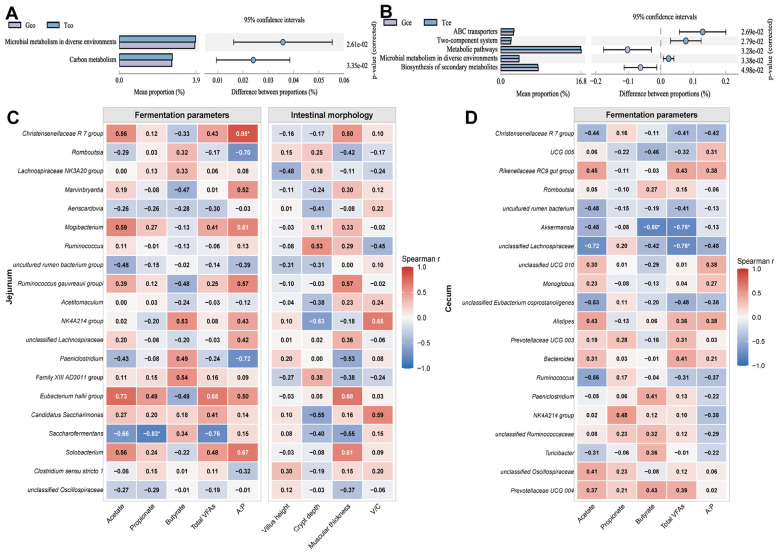
KEGG functional differences and genus-level microbial associations in intestinal segments. **(A, B)** Differential KEGG level 3 functional abundance in the cecum and colon, respectively. Values indicate BH-adjusted *P* values. **(C)** Spearman correlation heatmap between the top 20 jejunal genera and fermentation parameters and intestinal morphology. **(D)** Spearman correlation heatmap between the top 20 cecal genera and fermentation parameters. Values in panels C and D indicate Spearman's correlation coefficients (*r*, −1 to 1), and significant correlations are marked with asterisks. * indicates BH-adjusted *P* < 0.05.

PICRUSt2-based KEGG level 3 functional prediction showed that the top 15 pathways varied among intestinal segments and between breeds, mainly involving membrane transport, nucleotide metabolism, energy metabolism, and translation (Supplementary Figure 2A). Segment-specific comparisons indicated that functional differences were mainly detected in the cecum and colon. In the colon, microbial metabolism in diverse environments and carbon metabolism were higher in Tianhua mutton sheep than in Gansu Alpine fine-wool sheep, while in the cecum, metabolic pathways and biosynthesis of secondary metabolites were higher in Gansu Alpine fine-wool sheep, and microbial metabolism in diverse environments, ABC transporters, and two-component system were higher in Tianhua mutton sheep (Figures 6A, B). All the above differences were statistically significant (*P* < 0.05). Spearman correlation heatmaps were used to explore associations between the top 20 genera and phenotypic traits in different intestinal segments. In the small intestine, genus-level microbes were correlated with both fermentation parameters and intestinal morphology, whereas in the cecum and colon, they were correlated with fermentation parameters. In the ileum, *Lachnospiraceae_NK3A20_group* was positively correlated with propionate, and *Christensenellaceae_R_7_group* was positively correlated with propionate and total SCFA (Supplementary Figure 2F). In the jejunum, *Christensenellaceae_R_7_group* was positively correlated with A:P, whereas *Saccharofermentans* was negatively correlated with propionate (Figure 6C). In the cecum, *Akkermansia* was negatively correlated with butyrate and total SCFA, and *unclassified_Lachnospiraceae* was negatively correlated with total SCFA (Figure 6D). All the above correlations were statistically significant (*P* < 0.05).

## 4 Discussion

The Qinghai-Tibetan Plateau presents a unique ecological context for studying ruminant adaptation, characterized by hypoxic conditions, low temperatures, and seasonal variations in forage availability (Liu et al., 2022). This study provides a comprehensive examination of intestinal morphology, fermentation characteristics, and microbial adaptations in two distinct sheep breeds, Tianhua mutton sheep and Gansu Alpine fine-wool sheep, which have undergone long-term adaptation to similar high-altitude conditions but through different breeding trajectories (Chang et al., 2020; Ma et al., 2025; Li et al., 2024). Given the central role of the small intestine in nutrient digestion and absorption, HE staining was conducted in the duodenum, jejunum, and ileum to evaluate mucosal histomorphology, whereas SCFA profiles and bacterial communities were assessed across five intestinal segments, including the duodenum, jejunum, ileum, cecum, and colon.

The histological examination revealed substantial differences in small-intestinal morphology between the two breeds that may underlie their different digestive characteristics. Tianhua mutton sheep exhibited significantly lower crypt depth in the duodenum and ileum along with a higher villus height-to-crypt depth ratio compared to Gansu Alpine fine-wool sheep. These morphological characteristics suggest several functional advantages for Tianhua mutton sheep. The reduced crypt depth may indicate a lower epithelial cell turnover rate, potentially translating to reduced energy expenditure on tissue maintenance (Rubin and Levin, 2016). The higher V/C ratio provides increased surface area for nutrient absorption, representing an optimization for nutrient extraction from grazing forage resources (Casas et al., 2020). The muscular layer thickness showed a segment-specific pattern, with Gansu Alpine fine-wool sheep having a thicker muscular layer in the duodenum and Tianhua mutton sheep having a thicker muscular layer in the ileum. Differences in muscular layer thickness may contribute to variation in intestinal motility, digesta mixing and transit, thereby affecting nutrient exposure to the absorptive surface and intestinal functional efficiency (Martel et al., 2022). These findings suggest targeted structural differences among small-intestinal regions, possibly reflecting differential functional requirements along the digestive pathway.

The SCFAs profiles indicate that breed-related differences in intestinal fermentation were segment-dependent rather than confined to a single intestinal region. In the duodenum and jejunum, higher levels of acetic acid, propionic acid and Total SCFA in Tianhua mutton sheep suggest greater availability of fermentation-derived metabolites in proximal intestinal contents, which may support energy acquisition under grazing conditions (Zhou et al., 2019). The increased A:P ratio in the duodenum further indicates a shift in the balance between acetate- and propionate-related fermentation products. Because acetate is an important energy substrate for peripheral tissues and is involved in lipid metabolism, while propionate is closely associated with gluconeogenesis and glucose homeostasis, the simultaneous elevation of acetate, propionate, Total SCFA and A:P suggests a more active and acetate-oriented SCFAs supply pattern rather than a single fermentation pathway shift (Dalile et al., 2019; Chen et al., 2022). In the hindgut, where the cecum and colon function as important post-ruminal fermentation sites, the higher cecal acetic acid, butyric acid and Total SCFA, together with higher colonic propionic acid and Total SCFA, suggest a stronger capacity for SCFAs production or retention in Tianhua mutton sheep (Jing et al., 2025). Butyrate is an important energy source for intestinal epithelial cells and contributes to barrier function, while cecal propionate and butyrate metabolism has been linked to energy regulation and environmental responses in sheep (Cheng et al., 2025; Li et al., 2025). Thus, the SCFAs results suggest that Tianhua mutton sheep may have a more favorable and segment-specific microbial fermentation profile, which could partly contribute to more efficient energy acquisition and utilization under grazing conditions.

The comprehensive analysis of gut microbiota revealed clear breed- and segment-associated differences in microbial community structure. The sequencing quality assessment, including Good's Coverage values, supported the interpretation of dominant bacterial community patterns between breeds and intestinal segments. Tianhua mutton sheep exhibited higher microbial richness (Ace index) particularly in the jejunum, cecum, and colon, suggesting development of a more diverse and potentially more stable microbial community. Enhanced microbial diversity is generally associated with greater functional redundancy and ecosystem resilience, which could be advantageous in high-altitude environments with seasonal fluctuations in forage quality and availability (Lagkouvardos et al., 2019). The clear separation in beta diversity between breeds demonstrates the strong influence of host genetics on microbial community assembly, overriding shared environmental factors (Yang et al., 2022). This finding emphasizes the strength of host genetic control in determining microbial composition and supports the concept of co-adaptation between host and microbiome in response to selective pressures (Zhang et al., 2023; Zhen et al., 2023). The consistent enrichment of Firmicutes and *Christensenellaceae_R_7_group* in Tianhua mutton sheep across intestinal segments represents a hallmark of their microbial ecology. Firmicutes are particularly efficient in plant fiber degradation and energy extraction, consistent with the breed's selection for meat production traits requiring efficient nutrient utilization (Waters and Ley, 2019; Zhou et al., 2022). Many Firmicutes species specialize in breaking down recalcitrant plant fibers and producing short-chain fatty acids, making them crucial for energy extraction from forage-based diets characteristic of high-altitude grazing systems. *Christensenellaceae*, a heritable taxon frequently associated with healthy metabolic phenotypes, may contribute to improved energy metabolism and nutrient partitioning in Tianhua mutton sheep. The spatial organization of microbial communities along the intestinal tract showed consistent patterns between breeds, with clear separation between small intestinal and large intestinal samples, indicating conservation of fundamental niche specialization patterns despite taxonomic differences.

In contrast, Gansu Alpine fine-wool sheep exhibited a microbiota characterized by higher abundances of Bacteroidota, Verrucomicrobiota, and *Romboutsia*, including *Akkermansia*. While these taxa have diverse functions, their enrichment may reflect an alternative microbial strategy adapted to different host priorities. The differential abundance of mucin-degrading *Akkermansia* in Gansu Alpine fine wool sheep may indicate distinct host-mucosa relationships and alternative energy harvesting strategies (Cani et al., 2022). These breed-specific signatures exemplify how artificial selection for different production traits can shape distinct microbial ecosystems through host-mediated selection pressures (Sbierski-Kind et al., 2022; Theelen et al., 2023). The segment-specific analysis revealed additional layers of complexity, with particular enrichment of beneficial taxa in different intestinal regions of Tianhua mutton sheep, suggesting spatially organized functional specialization within their microbial ecosystem that supports efficient nutrient extraction and utilization.

The predicted functional profiles and correlation analyses provided additional clues for understanding breed-related differences in intestinal microbial function and fermentation traits. KEGG level 3 prediction showed that functional differences were mainly concentrated in the cecum and colon, which is consistent with the greater microbial complexity and fermentation activity of the hindgut. In Tianhua mutton sheep, the enrichment of microbial metabolism in diverse environments, ABC transporters and two-component systems in the cecum, together with higher carbon metabolism in the colon, suggests greater potential for substrate sensing, nutrient transport and metabolic conversion. Similar associations among dietary substrate availability, rumen or intestinal fermentation, microbial composition and functional pathways have also been reported in sheep and cattle, supporting the biological relevance of these predicted functions (Chen et al., 2024; Gao et al., 2024; Jing et al., 2025). These functional features may help explain the higher SCFA levels observed in several intestinal segments. The genus-level correlations further supported this interpretation. The positive associations of *Christensenellaceae_R_7_group* with propionate, Total SCFA and A:P, and of *Lachnospiraceae_NK3A20_group* with propionate, suggest that these taxa may participate in fermentation-related metabolic networks, consistent with the role of microbial cross-feeding in shaping gut metabolite production (Culp and Goodman, 2023). Previous sheep studies also reported that dietary or breed-related changes in gut microbial communities are closely associated with intestinal development, fermentation traits and microbial diversity, further supporting the potential contribution of these taxa to nutrient utilization (Chang et al., 2020; Zhou et al., 2022; Li et al., 2023). Notably, although intestinal morphology was included in the correlation analysis for the small-intestinal segments, significant associations were mainly detected between specific genera and fermentation parameters rather than histomorphological indices. This suggests that the observed morphological differences may not be directly explained by the dominant genera alone, but may also involve host genetic background, developmental regulation, local tissue responses, or microbial metabolites not fully captured by genus-level abundance. In the cecum, *Akkermansia* was negatively correlated with butyrate and Total SCFA, indicating that mucin-associated bacteria may be linked to a different fermentation pattern in Gansu Alpine fine-wool sheep. This interpretation is consistent with the known role of *Akkermansia* in mucin utilization and host–mucosa interactions (Cani et al., 2022). Because butyrate supports epithelial energy metabolism and intestinal barrier function, the associations between butyrate-related traits and specific genera may reflect differences in local intestinal functional status (Pearce et al., 2020; Martel et al., 2022). These results are also consistent with previous reports that Tianhua mutton sheep differ from Gansu Alpine fine-wool sheep in rumen development and production-related traits under grazing conditions (Li et al., 2024; Ma et al., 2025). Overall, these association-based findings provide functional clues for understanding breed-related differences in intestinal fermentation, but further metagenomic, metabolomic or multi-omics validation will be needed to clarify the underlying mechanisms (Zhang et al., 2024; Li et al., 2025).

## 5 Conclusion

This study demonstrates that Tianhua mutton sheep and Gansu Alpine fine-wool sheep have distinct intestinal characteristics under the same grazing conditions. The differences were reflected not only in small-intestinal morphology and SCFA profiles, but also in bacterial community composition, segmental microbial distribution, predicted functional pathways, and genus-level associations with fermentation traits. Compared with Gansu Alpine fine-wool sheep, Tianhua mutton sheep showed more favorable SCFA profiles in several intestinal segments and enrichment of microbial taxa and predicted functions related to fermentation and metabolic activity, particularly in the hindgut. These results highlight the importance of considering intestinal segment specificity when evaluating host–microbiota relationships in grazing sheep. The findings provide a useful basis for further studies on breed-related differences in intestinal function and microbial metabolism in Tianhua mutton sheep and Gansu Alpine fine-wool sheep.

## Data Availability

The raw data generated in this study can be found in the NCBI (https://www.ncbi.nlm.nih.gov/), accession PRJNA1080071.
