# Supplementary figures and images for: Segment-specific intestinal bacterial community structure is associated with short-chain fatty acid profiles and mucosal morphology in two high-altitude sheep breeds
Source: Front Microbiol. 2026 Jul 9;17:1873761. doi: 10.3389/fmicb.2026.1873761 (PMC13393221; doi:10.3389/fmicb.2026.1873761)

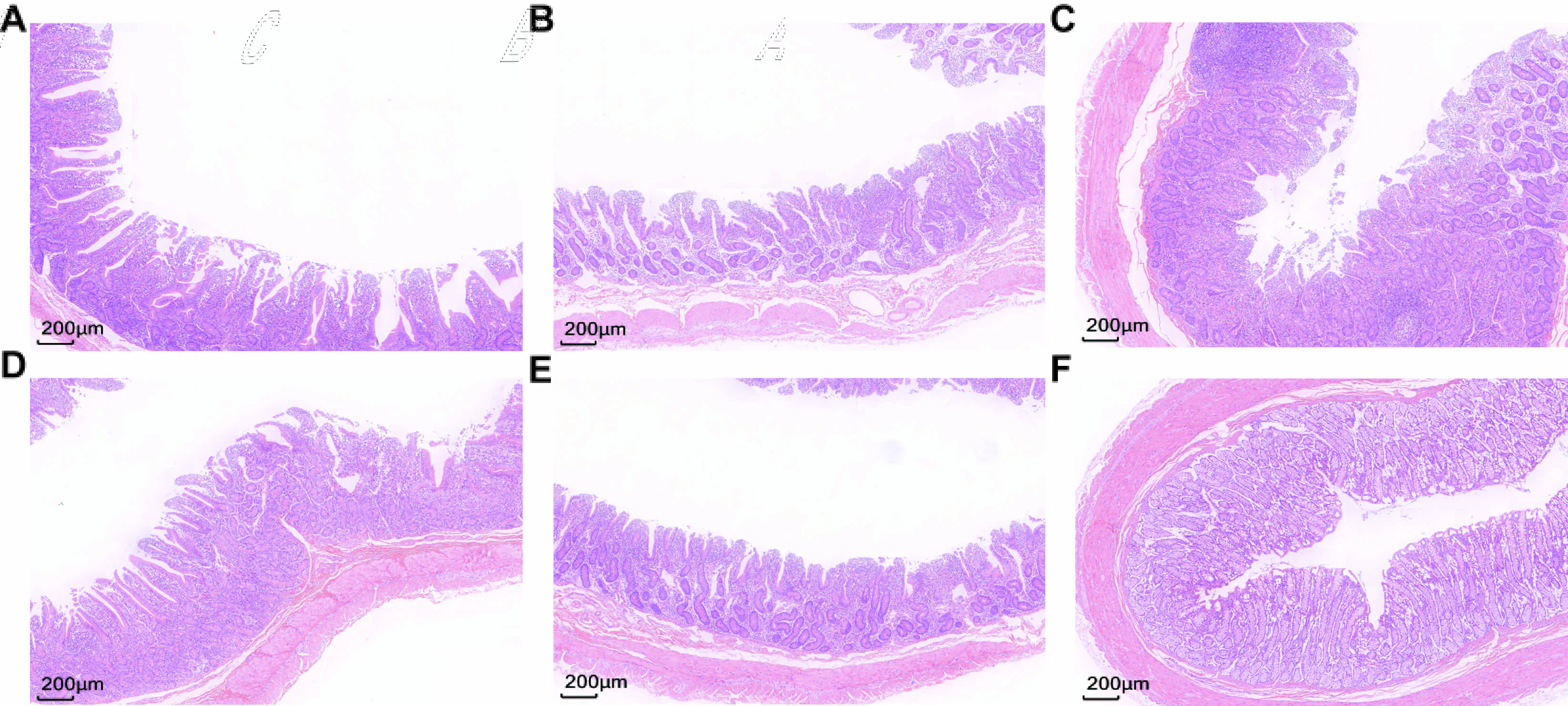

Supplement: Supplementary file 1 [file Image_1.jpeg]

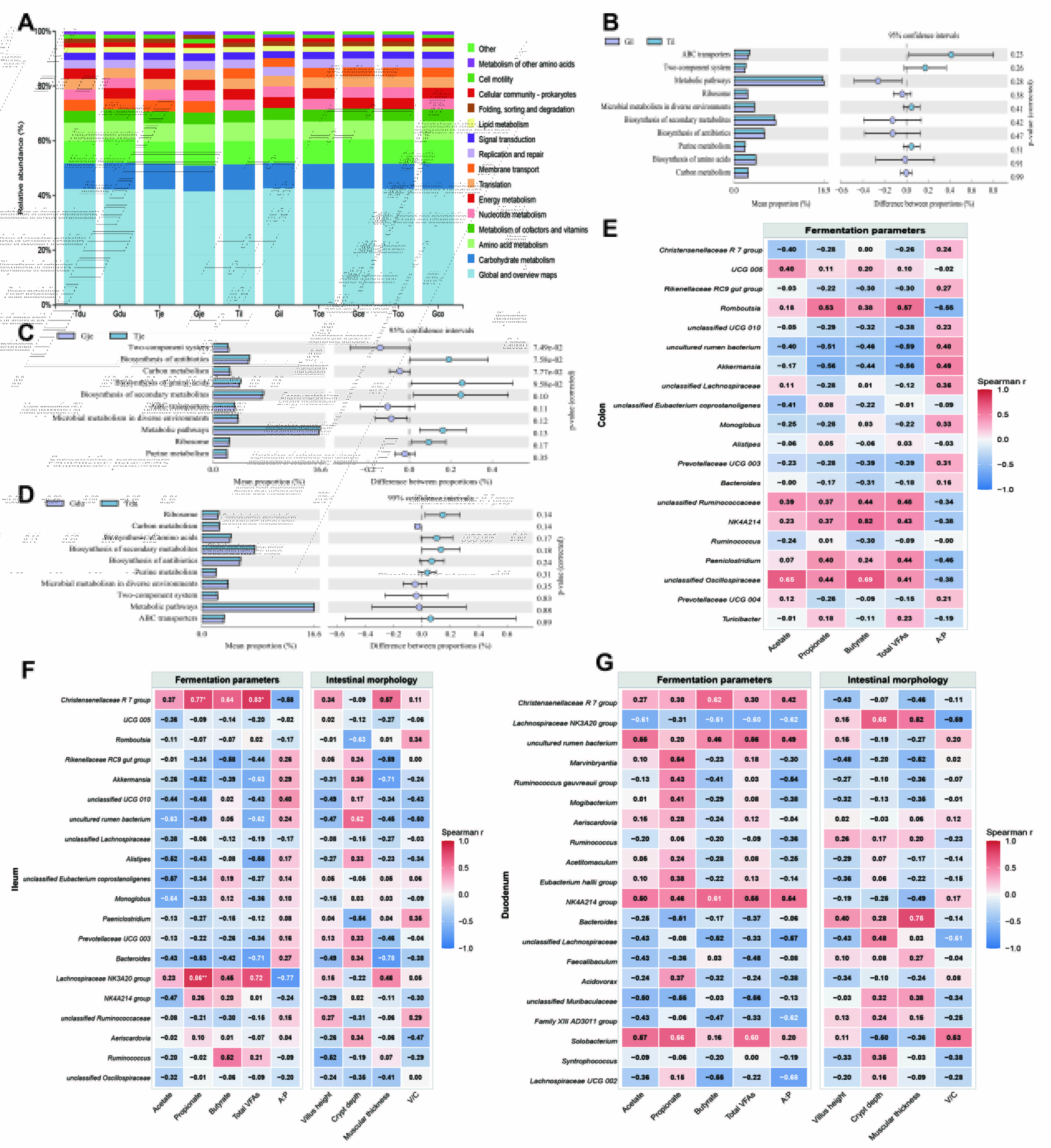

Supplement: Supplementary file 2 [file Image_2.jpeg]
