# Supplementary material for: Segment-specific intestinal bacterial community structure is associated with short-chain fatty acid profiles and mucosal morphology in two high-altitude sheep breeds
Source: Front Microbiol. 2026 Jul 9;17:1873761. doi: 10.3389/fmicb.2026.1873761 (PMC13393221; doi:10.3389/fmicb.2026.1873761)
Supplement: Supplementary file 3 [file Table_1.docx]

**Table S1 The routine nutritional content of two types of forage (mg/g DM)**

|  | CP | EE | ADF | NDF | Ash |
| --- | --- | --- | --- | --- | --- |
| *P. viviparum* | 101.5±0.90 | 22.65±0.36 | 313.63±4.04 | 360.17±5.96 | 63.32±0.11 |
| *E. nutans* | 92.18±1.08 | 23.00±0.98 | 434.20±3.28 | 697.85±6.38 | 53.14±0.12 |

CP: Crude protein; EE: Ether extract; ADF: acid detergent fiber; NDF: neutral detergent fiber; DM: dry matter
