# Supplementary material for: Segment-specific intestinal bacterial community structure is associated with short-chain fatty acid profiles and mucosal morphology in two high-altitude sheep breeds
Source: Front Microbiol. 2026 Jul 9;17:1873761. doi: 10.3389/fmicb.2026.1873761 (PMC13393221; doi:10.3389/fmicb.2026.1873761)
Supplement: Supplementary file 4 [file Table_2.docx]

**Table S2. Sample sequencing data processing results statistics (n=60)**

| Sample ID | Raw CCS | Clean CCS | Effective CCS | AvgLen(bp) | Effective(%) | Feature | Good’s Coverage |
| --- | --- | --- | --- | --- | --- | --- | --- |
| Gce1 | 40412 | 40400 | 33035 | 1446 | 81.75 | 4324 | 0.9459 |
| Gce2 | 36367 | 36345 | 31312 | 1446 | 86.10 | 4047 | 0.9420 |
| Gce3 | 35926 | 35915 | 29816 | 1448 | 82.99 | 4100 | 0.9363 |
| Gce4 | 37476 | 37451 | 32660 | 1447 | 87.15 | 4994 | 0.9257 |
| Gce5 | 36475 | 36458 | 31688 | 1447 | 86.88 | 4837 | 0.9293 |
| Gce6 | 34728 | 34721 | 30325 | 1445 | 87.32 | 4794 | 0.9273 |
| Gco1 | 33059 | 33058 | 27603 | 1448 | 83.50 | 4133 | 0.9285 |
| Gco2 | 35303 | 35285 | 29159 | 1447 | 82.60 | 3932 | 0.9379 |
| Gco3 | 39592 | 39571 | 32593 | 1448 | 82.32 | 4061 | 0.9492 |
| Gco4 | 39713 | 39697 | 32689 | 1445 | 82.31 | 4767 | 0.9317 |
| Gco5 | 33622 | 33606 | 28928 | 1449 | 86.04 | 4768 | 0.9191 |
| Gco6 | 33135 | 33103 | 28315 | 1446 | 85.45 | 4272 | 0.9257 |
| Gdu1 | 32847 | 32812 | 27272 | 1454 | 83.03 | 1755 | 0.9746 |
| Gdu2 | 34571 | 34552 | 29506 | 1455 | 85.35 | 2451 | 0.9650 |
| Gdu3 | 34494 | 34487 | 32832 | 1456 | 95.18 | 3091 | 0.9662 |
| Gdu4 | 37705 | 37628 | 34114 | 1456 | 90.48 | 1513 | 0.9771 |
| Gdu5 | 36043 | 36014 | 33370 | 1454 | 92.58 | 2044 | 0.9823 |
| Gdu6 | 32127 | 32100 | 31864 | 1454 | 99.18 | 1771 | 0.9829 |
| Gil1 | 40269 | 40253 | 34096 | 1447 | 84.67 | 4388 | 0.9433 |
| Gil2 | 35624 | 35611 | 31328 | 1445 | 87.94 | 3756 | 0.9468 |
| Gil3 | 32573 | 32561 | 27849 | 1447 | 85.50 | 3838 | 0.9379 |
| Gil4 | 32949 | 32933 | 28468 | 1447 | 86.40 | 4507 | 0.9232 |
| Gil5 | 37189 | 37176 | 32074 | 1447 | 86.25 | 4527 | 0.9352 |
| Gil6 | 32673 | 32663 | 27375 | 1445 | 83.78 | 4340 | 0.9173 |
| Gje1 | 35631 | 35602 | 34283 | 1451 | 96.22 | 2795 | 0.9716 |
| Gje2 | 31174 | 31120 | 29927 | 1444 | 96.00 | 1981 | 0.9788 |
| Gje3 | 40033 | 39975 | 32003 | 1444 | 79.94 | 1866 | 0.9736 |
| Gje4 | 33627 | 33602 | 31641 | 1451 | 94.09 | 2203 | 0.9727 |
| Gje5 | 35739 | 35718 | 31001 | 1454 | 86.74 | 2055 | 0.9763 |
| Gje6 | 34215 | 34060 | 32343 | 1441 | 94.53 | 2068 | 0.9810 |
| Tce1 | 38662 | 38638 | 33440 | 1446 | 86.49 | 5190 | 0.9240 |
| Tce2 | 35801 | 35797 | 31626 | 1448 | 88.34 | 5262 | 0.9216 |
| Tce3 | 37679 | 37636 | 33444 | 1446 | 88.76 | 5385 | 0.9185 |
| Tce4 | 37345 | 37336 | 32648 | 1448 | 87.42 | 5467 | 0.9159 |
| Tce5 | 38138 | 38094 | 34182 | 1447 | 89.63 | 5496 | 0.9200 |
| Tce6 | 34576 | 34563 | 29980 | 1446 | 86.71 | 4765 | 0.9250 |
| Tco1 | 38784 | 38771 | 33873 | 1447 | 87.34 | 5089 | 0.9240 |
| Tco2 | 34823 | 34800 | 30805 | 1449 | 88.46 | 5201 | 0.8880 |
| Tco3 | 37479 | 37445 | 34152 | 1446 | 91.12 | 5459 | 0.9181 |
| Tco4 | 33052 | 33042 | 29327 | 1448 | 88.73 | 5043 | 0.9152 |
| Tco5 | 41238 | 41214 | 36257 | 1447 | 87.92 | 5314 | 0.9292 |
| Tco6 | 33235 | 33179 | 29376 | 1446 | 88.39 | 4815 | 0.9027 |
| Tdu1 | 38322 | 38290 | 34064 | 1457 | 88.89 | 2690 | 0.9670 |
| Tdu2 | 39852 | 39839 | 38973 | 1453 | 97.79 | 2178 | 0.9830 |
| Tdu3 | 33173 | 33163 | 32145 | 1453 | 96.90 | 1983 | 0.9807 |
| Tdu4 | 29278 | 29260 | 28960 | 1456 | 98.91 | 2479 | 0.9728 |
| Tdu5 | 37877 | 37870 | 35990 | 1455 | 95.02 | 2476 | 0.9775 |
| Tdu6 | 40972 | 40958 | 38065 | 1456 | 92.90 | 2441 | 0.9760 |
| Til1 | 35808 | 35742 | 29813 | 1440 | 83.26 | 2446 | 0.9537 |
| Til2 | 40981 | 40969 | 34899 | 1452 | 85.16 | 2502 | 0.9687 |
| Til3 | 30239 | 30205 | 27823 | 1446 | 92.01 | 4969 | 0.9083 |
| Til4 | 34251 | 34211 | 30916 | 1447 | 90.26 | 5388 | 0.9072 |
| Til5 | 41133 | 41103 | 36533 | 1447 | 88.82 | 5528 | 0.9263 |
| Til6 | 36264 | 36246 | 31568 | 1447 | 87.05 | 5085 | 0.9180 |
| Tje1 | 41018 | 40985 | 33574 | 1450 | 81.85 | 2992 | 0.9562 |
| Tje2 | 33244 | 33234 | 28268 | 1455 | 85.03 | 3015 | 0.9435 |
| Tje3 | 40648 | 40628 | 33464 | 1455 | 82.33 | 2514 | 0.9683 |
| Tje4 | 34690 | 34680 | 31809 | 1453 | 91.70 | 3086 | 0.9600 |
| Tje5 | 37085 | 37063 | 30274 | 1455 | 81.63 | 2566 | 0.9626 |
| Tje6 | 40158 | 40127 | 32948 | 1450 | 82.05 | 2457 | 0.9679 |
